# Vegetation index trends under contrasting land-use occupation in Brazilian savannas: a paradox between the established region and the agricultural frontier

**DOI:** 10.1007/s10661-026-15637-y

**Published:** 2026-07-01

**Authors:** Yan Breno Azeredo Gomes da Silva, Lênio Soares Galvão, Fábio Marcelo Breunig, Lucas Batista de Oliveira

**Affiliations:** 1 Divisão de Observação da Terra e Geoinformática (DIOTG), Programa de Pós-Graduação em Sensoriamento Remoto, Coordenação-Geral de Ciências da Terra (CGCT), Instituto Nacional de Pesquisas Espaciais (INPE), Avenida dos Astronautas, 1758, Jardim da Granja, 12227-010 SP, Brasil; 2 Departamento de Geografia, LAPE (Laboratório de Análise de Padrões Espaciais), Universidade Federal do Paraná (UFPR), Sala 202, Edifício João José Bigarella, Curitiba, 80060-000 PR Brasil; 3 Instituto de Matemática e Estatística, Universidade Federal Fluminense (UFF), Niterói, 24210-201 RJ Brasil

**Keywords:** NDVI, MODIS, Cerrado, MATOPIBA, Monitoring, Remote sensing

## Abstract

Long-term trajectories of the Normalized Difference Vegetation Index (NDVI) provide insights into ecosystem changes associated with vegetation productivity and land degradation. However, their interpretation may depend on land-use history. Here, we analyzed NDVI trajectories from Moderate Resolution Imaging Spectroradiometer (MODIS) data (2000–2022) across two contrasting regions in the Brazilian Cerrado: an established agricultural region and an expanding frontier. We evaluated whether NDVI trends differ between regions as a function of land-use dynamics and environmental/climatic conditions. NDVI trajectories were derived using Trends.Earth and correlated with MODIS Gross Primary Production (GPP). Common and region-specific trend drivers were analyzed using binary logistic regression and Wald-type Z tests. Results revealed a remote-sensing paradox. Despite pronounced differences between regions, the primary common driver of NDVI decline was the native savanna conversion to croplands and pastures. Paradoxically, the established agricultural region, where most clearing predated satellite observations, was the most stable, showing the smallest pixel proportion of NDVI decrease (7%) and the highest proportion of increase (52%). In contrast, the agricultural frontier, where native vegetation dominates and clearing largely coincided with satellite observations, showed greater NDVI decline (16%) and smaller increases (37%). Positive NDVI–GPP relationships were weaker in the established region. Region-specific drivers included precipitation, soil sand content, and fire frequency. Longer agricultural use duration contributed to NDVI increases, likely reflecting management practices that mask underlying degradation. Our findings indicate that NDVI-based assessments of land degradation in the Cerrado must consider early land-use history and agricultural management relative to the period of satellite observations.

## Introduction

Brazilian savannas comprise a biome locally known as the Cerrado, characterized by mosaics of grasses, shrubs, and woodlands, and covering approximately 2,040,000 km^2^ (Ferreira et al., 2011; Sano et al., 2019). Ecologically, the Cerrado is recognized as a global biodiversity hotspot due to its high levels of endemism and species richness (Klink & Machado, 2005; Myers et al., 2000). From an agricultural perspective, the biome plays a central role in Brazil’s economy, representing the country’s largest grain-producing region, particularly for soybean and corn, and a major center of livestock production (Beuchle et al., 2015; Embrapa, 2005; IBGE, 2025).

Land-use occupation across the Cerrado is spatially heterogeneous and remains poorly understood at the biome scale. Over recent decades, croplands and pastures have expanded outward from long-established agricultural areas in the central and southern portions of the biome toward a more recent agricultural frontier in its northern sector (Souza et al., 2020). This frontier is commonly referred to as MATOPIBA, an acronym formed from the states of Maranhão, Tocantins, Piauí, and Bahia. MATOPIBA represents the most recent, and likely the final, major agricultural frontier for cropland and pasture expansion within the Cerrado (Graesser et al., 2015; Rausch et al., 2019).

In the established agricultural region, the progressive adoption of crop and soil management practices over the past six decades, including soil acidity correction, fertilization, the development of aluminum-tolerant crop varieties, and the widespread implementation of no-till systems, has enabled large-scale agricultural expansion at the expense of native savanna vegetation (Muhie, 2022; Souza et al., 2024). In the newest agricultural frontier (MATOPIBA), native vegetation predominates but the rates of clearing have accelerated in recent years and have surpassed contemporary rates of tropical forest conversion in the Amazon (Bispo et al., 2023; Souza et al., 2020; Zalles et al., 2019). At the biome scale, it is estimated that approximately half of the original native vegetation of the Cerrado has already been converted to anthropogenic land use (Bispo et al., 2020; Sano et al., 2010; Silva et al., 2025).

In the context of rapid and spatially uneven land-use and land-cover (LULC) changes, the analysis of long-term vegetation dynamics is very important. Trajectory analysis of the Normalized Difference Vegetation Index (NDVI), derived from Moderate Resolution Imaging Spectroradiometer (MODIS) data acquired since the launch of the Terra satellite in December 1999, offers a means to detect long-term ecosystem changes potentially associated with vegetation productivity and land degradation (Paredes-Trejo et al., 2023). NDVI is the most widely used vegetation index and has been adopted across numerous studies and applications (e.g., Dastigerdi et al., 2024; Sayedzadeh et al., 2024; Shrestha et al., 2024). Compared with other greenness-sensitive indices, NDVI is less affected by shadows from variable solar and terrain illumination conditions and by the viewing geometry of large field-of-view sensors such as MODIS (Galvão et al., 2024; Matsushita et al., 2007). It does not also saturate under sparse vegetation conditions commonly found in most savanna physiognomies.

Open-source tools such as Trends.Earth, which rely on annual integrals of NDVI to quantify long-term trends of increase, stability, or decline, have facilitated the assessment of LULC impacts on ecosystems at broad spatial scales (Conservation International, 2022; Schillaci et al., 2023). For instance, long-term declines in MODIS NDVI at the pixel level may indicate reduction in vegetation productivity and the expansion of degraded lands. Despite this potential, studies applying MODIS NDVI time-series analysis in the Cerrado remain limited. Existing research has primarily focused on pasture degradation (Pereira et al., 2018) or on localized assessments within MATOPIBA (Souza et al., 2020). Moreover, factors controlling the NDVI trajectories across the biome have received little attention, with most analyses restricted to small study areas rather than addressing the Cerrado as an integrated system. As emphasized by Silva et al. (2025), the drivers of NDVI variability are likely to vary across regions of the Cerrado biome characterized by distinct land-use histories and environmental/climatic conditions, indicating the need for further investigations. A better understanding of such dynamics is important for defining initial and transition states in vegetation monitoring (Jones et al., 2023).

In this context, the coexistence of a long-established agricultural region and an actively expanding agricultural frontier within the Cerrado provides an interesting experimental design for examining NDVI trends over a fixed period of satellite observation (2000–2022) and under contrasting patterns of land-use occupation. In the established region, where native savanna vegetation has been largely converted into croplands and pastures, MODIS may capture patterns of vegetation productivity and land degradation resulting from LULC changes that occurred more than 30 years prior to the onset of satellite observations at the end of 1999. In contrast, in the newer agricultural frontier of the Cerrado (MATOPIBA region), LULC changes are predominantly occurring contemporaneously with the period of MODIS observations. Therefore, the validity of NDVI as a proxy for vegetation productivity and land degradation in the Cerrado biome may depend on regional land-use dynamics relative to the period of satellite observation. Based on this premise, we hypothesize that NDVI trajectories in the Cerrado have distinct patterns and different primary drivers under contrasting regions of land-use history.

The objective of this study is to investigate whether distinct patterns and drivers of long-term NDVI variability emerge between the established agricultural region and the expanding agricultural frontier, reflecting their contrasting land-use dynamics during a fixed period of MODIS observation (2000–2022), and environmental/climatic differences. For this purpose, we pursue the following specific objectives: (i) to characterize the dynamics of native savanna vegetation replacement by croplands and pastures in each region; (ii) to detect NDVI trajectories of increase, decrease, and stability across regions, correlating the NDVI trends with MODIS Gross Primary Production (GPP) estimates derived from the MOD17A3HGF product; and (iii) to identify common and region-specific drivers of NDVI trajectories using binary logistic regression on variables describing LULC changes, crop and pasture use duration, fire frequency, precipitation, soil sand fraction, and topography, complemented by Wald-type Z tests.

## Methodology

**Fig. 1:**
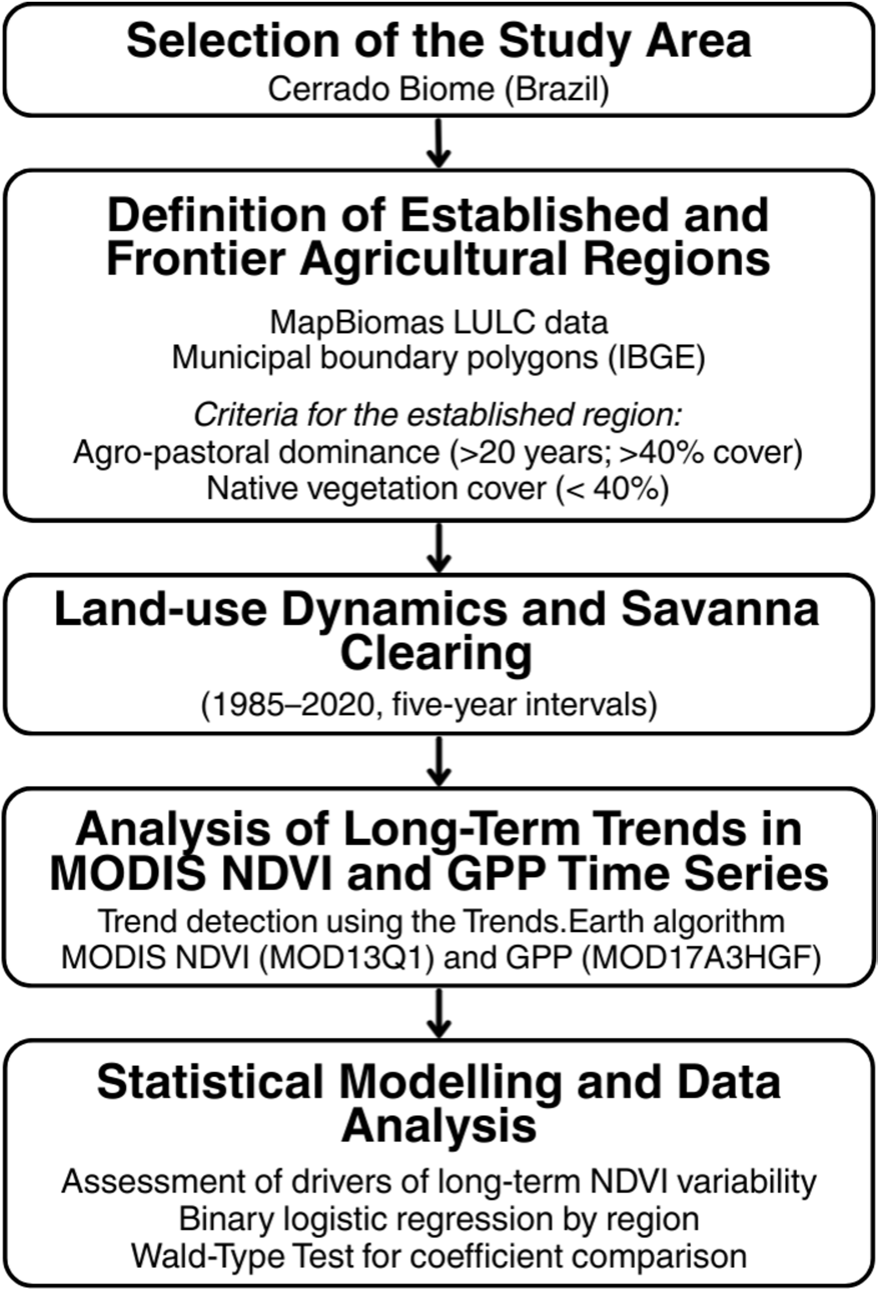
Synthesis of the methodological procedures adopted in this study

Figure 1 summarizes the methodological workflow of the investigation, including study area selection, definition of established and frontier agricultural regions in the Cerrado, analysis of land-use dynamics, assessment of long-term NDVI and GPP trends, and application of statistical models to investigate potential drivers of long-term NDVI trajectories.

### Selection of the study area and delineation of the regions

**Fig. 2:**
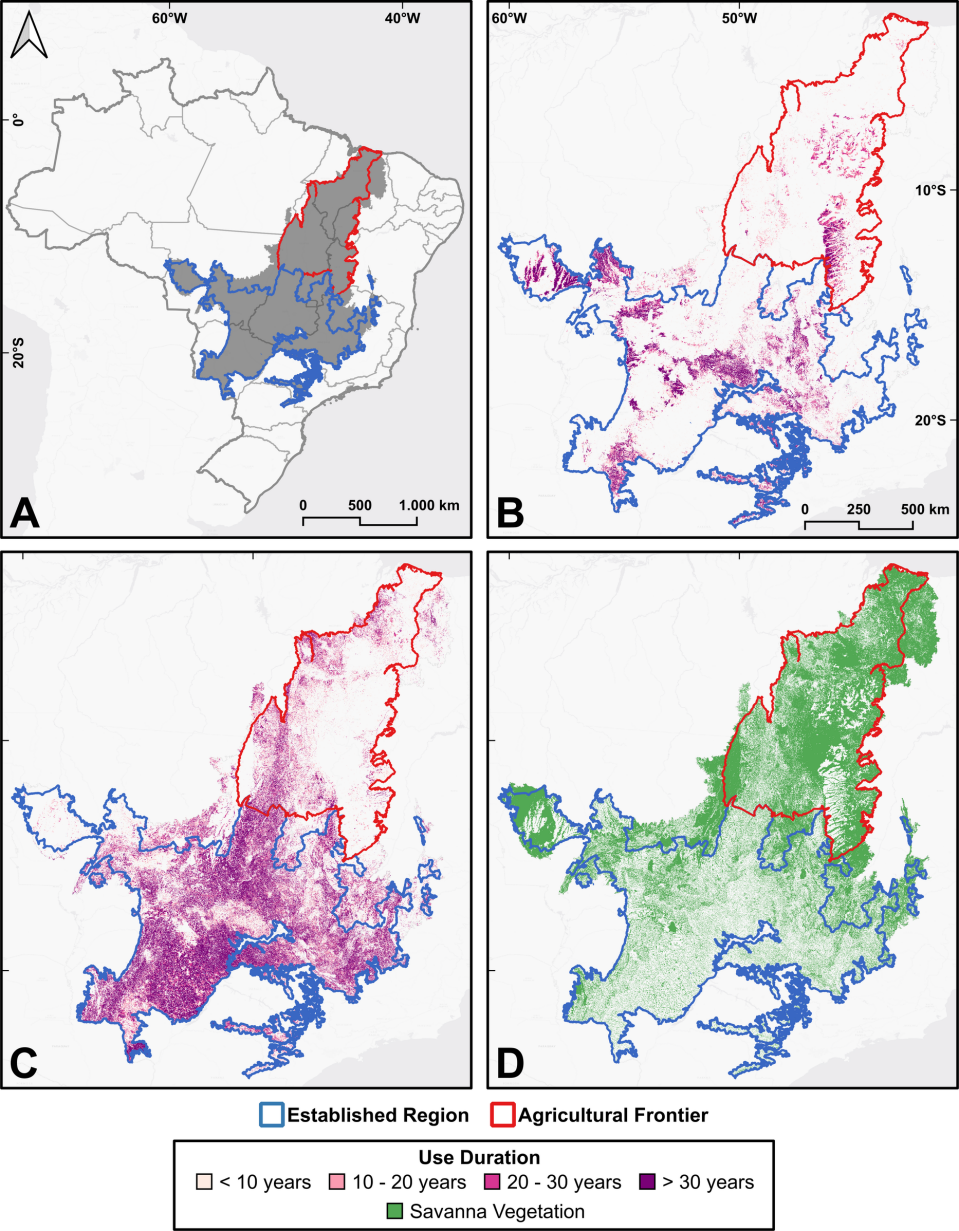
**A** Location of the Cerrado biome in Brazil. Continuous land-use duration for croplands and pastures is shown in **B** and **C**, respectively, classified into four temporal classes (<10, 10–20, 20–30, and >30 years of land occupation). The distribution of remaining native savanna vegetation in 2022 is shown in **D**. Blue and red outlines in (**A**–**D**) indicate the established agricultural region and the agricultural frontier (MATOPIBA region), respectively

The study area comprises the entire Brazilian savanna biome (Cerrado), which covers approximately 24% of Brazil and is the country’s second-largest biome after the Amazon (Fig. 2A). Within this biome, two contrasting regions were delineated to capture different stages of agricultural development: an established agricultural region and an expanding agricultural frontier. They are shown as blue (established agricultural region) and red (agricultural frontier) polygons in Figs. 2A–D.

The established agricultural region was delineated using three municipal-level criteria based on pixel proportions: (i) croplands with a use duration longer than 20 years and agricultural vegetation cover exceeding 40%; (ii) pasturelands with a use duration longer than 20 years and agricultural vegetation cover exceeding 40%; and (iii) native savanna with vegetation cover below 40%. LULC information was derived from the MapBiomas Project (Collection 10) (https://brasil.mapbiomas.org/), using quinquennial maps from 1985 to 2020 reclassified into savannas, pastures, crops, and “other” classes (e.g., urban areas). Indicators of cropland and pasture use duration were computed from these maps and used exclusively for regionalization purposes, while the 2022 land cover map was employed to estimate the area of remaining native savannas. Municipal boundaries were obtained from the Instituto Brasileiro de Geografia e Estatística (IBGE). The 20-year threshold ensured that the majority of LULC changes occurred prior to the MODIS NDVI analysis period (2000–2022).

The agricultural frontier was delineated using the official MATOPIBA boundary established by Decree No. 8,447 (May 6, 2015). Only the portion of MATOPIBA located within the Cerrado biome was considered, corresponding to approximately 86% of the total MATOPIBA area.

Compared to the expanding frontier, the established agricultural region is dominated by long-standing croplands (Fig. 2B) and pastures (Fig. 2C), with reduced and highly fragmented native savanna vegetation (Fig. 2D). Although the agricultural frontier also contains localized areas of long-established crops and pastures, these areas were retained within the frontier for further analyses because they are embedded in the spatial and functional context of recent agricultural expansion. Nonetheless, such localized areas represent only a small proportion of the total delimited frontier.

### Dynamics of savanna clearing and land occupation in the studied regions

The analysis of savanna replacement by croplands and pastures in both the established agricultural region and the agricultural frontier was based on 30-m spatial resolution LULC maps derived from the MapBiomas Project (Collection 10). Using the QGIS Geographic Information System (version 3.44), the areas (km^2^) occupied by the three land-cover classes (savannas, pastures, and croplands) were quantified at 5-year intervals from 1985 to 2020. For each interval, percentage rates of change in savanna area were also calculated.

This approach allowed for a consistent and comparable assessment of land-use dynamics between the two regions, with particular emphasis on the temporal patterns of savanna clearing and the expansion or contraction of cropland and pasture areas over time.

### Trend analysis of the MODIS NDVI and GPP time series

Long-term NDVI trends were assessed using the Trends.Earth algorithm, a freely available tool implemented within the QGIS environment that operates through remote access to the Google Earth Engine (GEE) platform (Conservation International, 2022). Annual NDVI integrals were computed from the MODIS MOD13Q1 product (Collection 6; 250 m spatial resolution), generating a continuous time series suitable for long-term trend analysis. Prior to trend estimation, NDVI values were corrected for climatic variability using the Rain Use Efficiency (RUE) approach, which was applied uniformly across both regions to ensure comparability of the results. This step is essential for decoupling human-induced effects on land degradation and vegetation productivity from those driven by regional differences in rainfall (Wessels et al., 2007). In both regions, water is a strong limiting factor for vegetation development. Using Trends.Earth, RUE was computed as the ratio of the annual NDVI integral to annual precipitation from the Climate Hazards Group Infrared Precipitation with Stations (CHIRPS) dataset. Linear regression and the Mann–Kendall test (*p* < 0.05) were subsequently applied to the resulting time series.

Trends.Earth provides two complementary metrics for characterizing NDVI dynamics: Trajectory and State. The Trajectory metric captured long-term NDVI behavior over the 2000–2022 period, which was selected to maximize the temporal extent of the time series and ensure statistical robustness. This metric was obtained by fitting a linear regression to the annual NDVI integrals, with trend significance evaluated using the nonparametric Mann–Kendall test (*p* < 0.05). Based on this procedure, pixels were classified as exhibiting positive, negative, or non-significant trends, corresponding to NDVI increases, declines, or stability, respectively.

The State metric complemented the long-term trajectory analysis by identifying more recent trends in NDVI. By using this metric, we compared a recent period (2017–2022) with a reference period (2000–2016). For each interval, mean annual NDVI integrals were calculated and classified into ten percentile classes, ranging from 1 (lowest values) to 10 (highest values). The difference between percentile classes assigned to the two periods was then computed, with values ≤ −2 interpreted as indicative of potential recent NDVI decline and values ≥ +2 as indicative of potential NDVI increase. The use of a ±2 threshold, adopted as the standard default in Trends.Earth, allows robust changes in biological productivity to be distinguished from minor interannual variability or climatic noise, which are interpreted as indicative of stability. The Trajectory and State metrics were subsequently combined into a single Aggregated NDVI Trend Indicator, synthesizing information on NDVI trajectories into three categories: decline, stability, and increase.

To relate NDVI trends to vegetation productivity, linear trends in GPP were estimated using a methodology consistent with that applied to NDVI. Annual GPP data from the MODIS MOD17A3HGF product (version 6.1; 500 m spatial resolution) were obtained for the 2000–2022 period and resampled to 250 m to match the NDVI spatial resolution. Pixelwise linear regressions were fitted to the GPP time series, with trend significance assessed using the Mann–Kendall test. Finally, GPP trend slopes were correlated with NDVI trends using an automated random sampling procedure that selected 500 pixels per region. This sample size was defined to ensure a margin of error below 5%, while preserving adequate representativeness within the region. Additionally, restricting the number of sampled pixels minimized spatial autocorrelation, thereby enhancing the robustness and independence of the correlation analysis.

### Statistical analysis and modelling

The statistical analysis was conducted in two sequential stages. First, binary logistic regression models were applied to identify the main factors influencing NDVI trajectories in both the established agricultural region and the agricultural frontier. Second, model coefficients were systematically compared between regions using Wald-type Z tests to assess whether individual factors exert common or region-specific effects on NDVI trends. Prior to describing these analytical procedures in detail, the categorical and discrete variables included in the models are presented and characterized.

#### Selection of potential factors explaining long-term NDVI trends

Table 1 summarizes the categorical and discrete variables selected for the statistical modeling of potential drivers of NDVI trajectories. LULC data were obtained from the MapBiomas Project at 30-m spatial resolution. Following Silva et al. (2025), quinquennial LULC maps were produced for the period 1985–2020, considering the three classes: savannas, pastures, and croplands. An additional map corresponding to 2022, marking the end of the study period, was also obtained. Based on this time series, indicators of Crop Use Duration and Pasture Use Duration were derived. As illustrated in Fig. 2, four classes of continuous crop and pasture use were defined: (a) less than 10 years, (b) 10–20 years, (c) 21–30 years, and (d) more than 30 years.

**Table 1:** Potential drivers of long-term MODIS NDVI trends in the established and agricultural frontier regions selected for binary logistic regression. Categorical (C) and discrete (D) types of variables are indicated

Variable	Description	Spatial resolution (m)	Type	Source
Land Use and Land Cover (LULC)	Savannas, pastures and crops (5-year maps from 1985 to 2020; and 2022)	30	C	MapBiomas Project
Crop Use Duration	Duration classes of crop use (<10, 10–20, 20–30, >30 years)	30	C	MapBiomas Project; this study
Pasture Use Duration	Duration classes of pasture use (<10, 10–20, 20–30, >30 years)	30	C	MapBiomas Project; this study
Land Use and Land Cover (LULC) Change	Land-use and land-cover changes, including preserved areas of native savannas or their conversion into crops and pastures	30	C	MapBiomas Project
Elevation	Digital Elevation Model (m)	30	D	SRTM (TOPODATA)
Slope	Slope classes (flat, gently undulating, undulating, strongly undulating, hilly, and very hilly)	30	D	SRTM (TOPODATA)
Sand Content	Soil sand content (g kg⁻^1^)	90	D	PronaSolos (Embrapa)
Precipitation	Annual precipitation (mm year⁻^1^)	5000	D	CHIRPS
Fire Frequency	Fire frequency classes (low, moderate, and high)	500	D	MODIS (MCD64A1)

Topographic variables, including elevation and slope, were obtained from the Brazilian Geomorphometric Database (TOPODATA), developed by the Instituto Nacional de Pesquisas Espaciais (INPE) and derived from Shuttle Radar Topography Mission (SRTM) data. Slope values were classified into six categories: flat (0–3%), gently undulating (3–8%), undulating (8–20%), strongly undulating (20–45%), hilly (45–75%), and very hilly (>75%). Soil sand content was obtained from the National Program for Soil Survey and Interpretation in Brazil (PronaSolos), coordinated by the Empresa Brasileira de Pesquisa Agropecuária (Embrapa), which provides estimates of subsurface (15–30 cm) sand content (g kg⁻^1^) at a spatial resolution of 90 m (Table 1).

Climatic and disturbance-related variables were also included in the analysis. Precipitation data were derived from the CHIRPS dataset, which integrates satellite observations and meteorological station data at a spatial resolution of 5 km. Annual total precipitation for the 2000–2022 period was extracted for both regions, and mean precipitation over this interval was calculated using QGIS. Fire frequency was estimated using the MODIS MCD64A1 burned area product, which detects fire occurrence based on vegetation indices derived from shortwave infrared (SWIR) bands at a spatial resolution of 500 m. For each pixel, we computed the number of fire events recorded over the study period and subsequently classified it into three categories based on terciles of the observed frequency distribution: low, moderate, and high fire frequency.

For the statistical modeling, all predictor variables were harmonized to the 250 m resolution of the NDVI product. Higher-resolution variables (LULC, topography, and soils; 30–90 m) were aggregated using the dominant class (categorical) or spatial mean (continuous), whereas lower-resolution variables (fire and precipitation; 0.5–5 km) were resampled using bilinear interpolation (continuous) or dominant class (categorical).

#### Binary logistic regression by region

The dependent variable was derived from the Aggregated NDVI Trend Indicator obtained from the long-term MODIS NDVI analysis and subsequently recoded as a binary variable distinguishing pixels exhibiting long-term NDVI decline from those without decline. Given this binary outcome, a binomial generalized linear model with a logit link function (logistic regression) was employed. This method estimates the probability of occurrence versus non-occurrence of a categorical binary event, that is, the probability of the event of interest and its complement (Harrell, 2015).

Binary logistic regression models were fitted separately for the established agricultural region and the expanding frontier. For each region, a random sample of pixels was drawn by combining the Aggregated NDVI Trend Indicator with the auxiliary variables listed in Table 1, forming two groups: pixels with long-term NDVI decline (coded as 1) and pixels without decline (coded as 0). Sample size was determined using the formula for proportions, as the variance of the categorical dependent variable was not known a priori.

Following Silva et al. (2025), a sampling proportion of 50% was adopted to maximize sample size, assuming a 95% confidence level and a 2% margin of error. This procedure resulted in 2400 observations per group for each region. All regression analyses were conducted in R (version 4.5.0; R Core Team^2025^). Multicollinearity among predictor variables was evaluated using the Variance Inflation Factor (VIF). Model performance was assessed using the Receiver Operating Characteristic (ROC) curve, with the Area Under the Curve (AUC) as the primary measure of discrimination, complemented by confusion matrices and associated accuracy metrics.

#### Wald-type test for equality of coefficients from logistic regression models

After fitting the logistic regression models for each region, the estimate coefficients were compared between the established agricultural region and the agricultural frontier using a Wald-type Z test. This procedure evaluates the null hypothesis that coefficients obtained from independent models are equal, explicitly accounting for the uncertainty associated with each estimate (Clogg et al., 1995). As discussed by Hosmer et al. (2013) and Harrell (2015), this test is appropriate for generalized linear models, including binary logistic regression, provided that model specifications are correct, estimate coefficients are independent, standard errors are reliable, and the compared predictors are expressed on the same scale.

By directly testing differences in the magnitude of the coefficients, this approach avoids potentially misleading interpretations based solely on individual significance levels or coefficient signs. Moreover, it is particularly well suited for contrasting regional analyses, such as those performed in this study, as it enables transparent and reproducible comparisons of predictor effects across regions (Gelman & Stern, 2006). Accordingly, the Wald-type test allows a robust assessment of whether the effects of explanatory variables on NDVI trajectories differ statistically between the established agricultural region and the agricultural frontier.

## Results

### Dynamics of savanna conversion into croplands and pastures by region

**Fig. 3:**
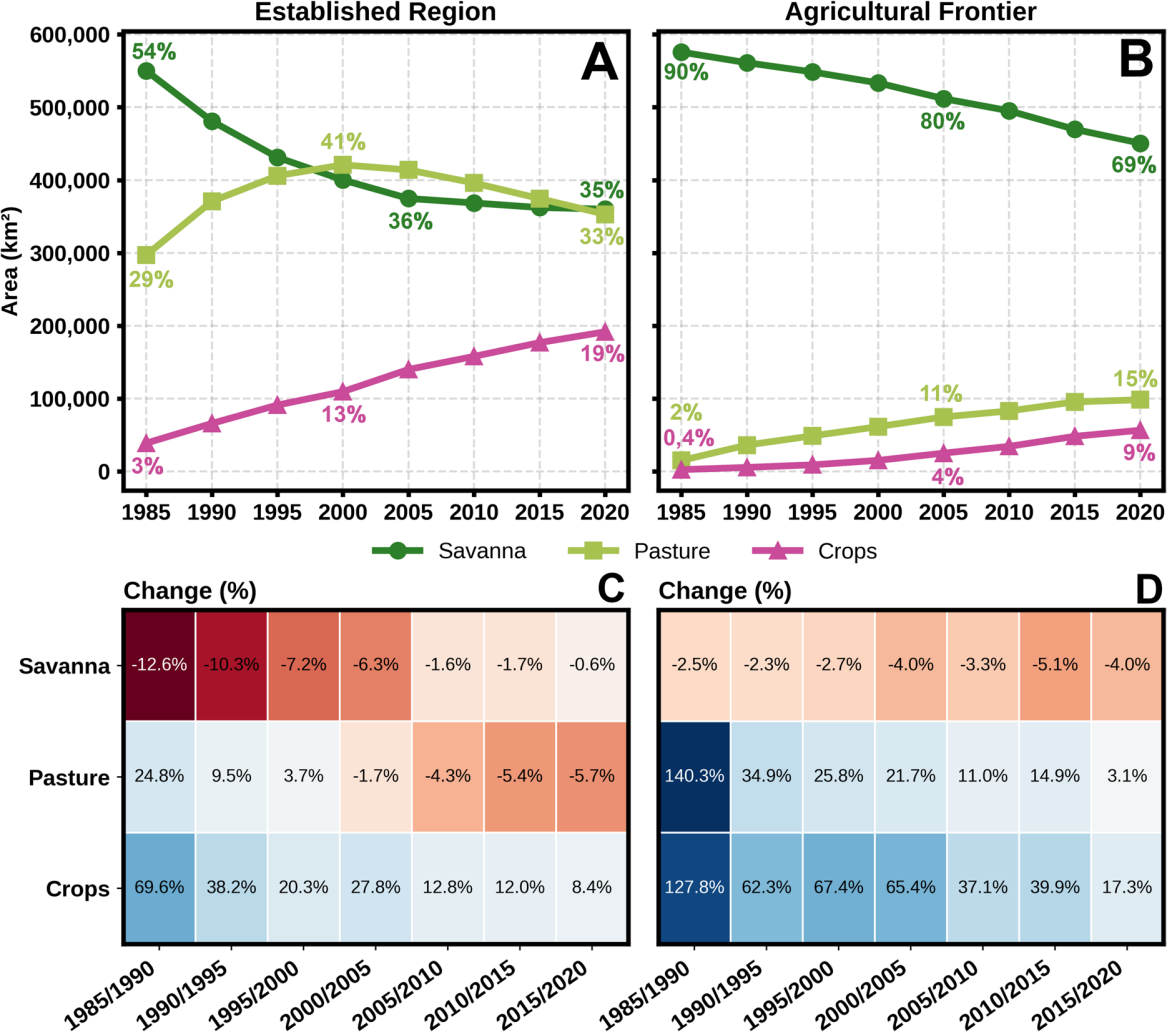
Dynamics of the native savanna conversion into croplands and pastures in **A** the established agricultural region and **B** the agricultural frontier of the Cerrado biome (1985–2020). Percentage rates of change over quinquennial intervals (1985–1990 to 2015–2020), calculated from the relative variation between consecutive periods, are shown for each region in **C** and **D**. The heatmap illustrates the magnitude and direction of changes for each land cover type. Reddish shades represent negative values (reduction in land-cover area), and bluish shades represent positive values (expansion)

Contrasting patterns of land-use occupation were observed between the established agricultural region and the emerging frontier from 1985 to 2020 (Fig. 3). In the established region, native savanna area declined sharply from approximately 550,000 km^2^ (54%) in 1985 to less than 400,000 km^2^ (36%) in 2005, followed by a relative stabilization in clearing towards 2020 (Fig. 3A). Native vegetation was predominantly converted to pastures, whose area increased proportionally from 29% in 1985 to 41% in 2000, before declining to 33% in 2020 as new croplands expanded over former pasture areas (Fig. 3A). In contrast, land-use occupation in the agricultural frontier was characterized by a continuous reduction in native savanna cover from approximately 557,000 km^2^ (90%) in 1985 to 450,000 km^2^ (69%) in 2020, driven by the expansion of croplands (9% in 2020) and pastures (15%) (Fig. 3B). Thus, by 2020, native savanna accounted for 35% of the area in the established region (Fig. 3A) and 69% in the emerging frontier (Fig. 3B). The total cleared area of savannas from 1985 to 2020 in these regions (~1,007,000 km^2^) accounted for roughly 50% of the original vegetation cover of the Cerrado biome. Pasture areas surpassed crop areas in both regions.

As shown in the heatmap of Fig. 3C, the highest percentages of clearing in native savanna areas of the established region occurred before the period of MODIS data acquisition (1985 to 2000). Before the satellite launch at the end of 1999, the percentage of cleared savanna area ranged from −12.6% (1985–1990) to −7.2% (1995–2000), as indicated by the reddish shades in the first row of Fig. 3C. On the other hand, declines in pasture areas, reflecting cropland expansion, occurred during the period of MODIS operation, as shown by the shift from blue to red shades between 2000 and 2020 (second row in Fig. 3C). Unlike the established agricultural region, savanna conversion accelerated in the frontier during the MODIS data acquisition period, with percentage rates ranging from −4.0% to −5.1% (Fig. 3D). Across both regions, croplands exhibited higher percentage changes than pastures during the 1985–2020 period (Fig. 3C and D).

### Long-term MODIS NDVI and GPP trends

**Fig. 4:**
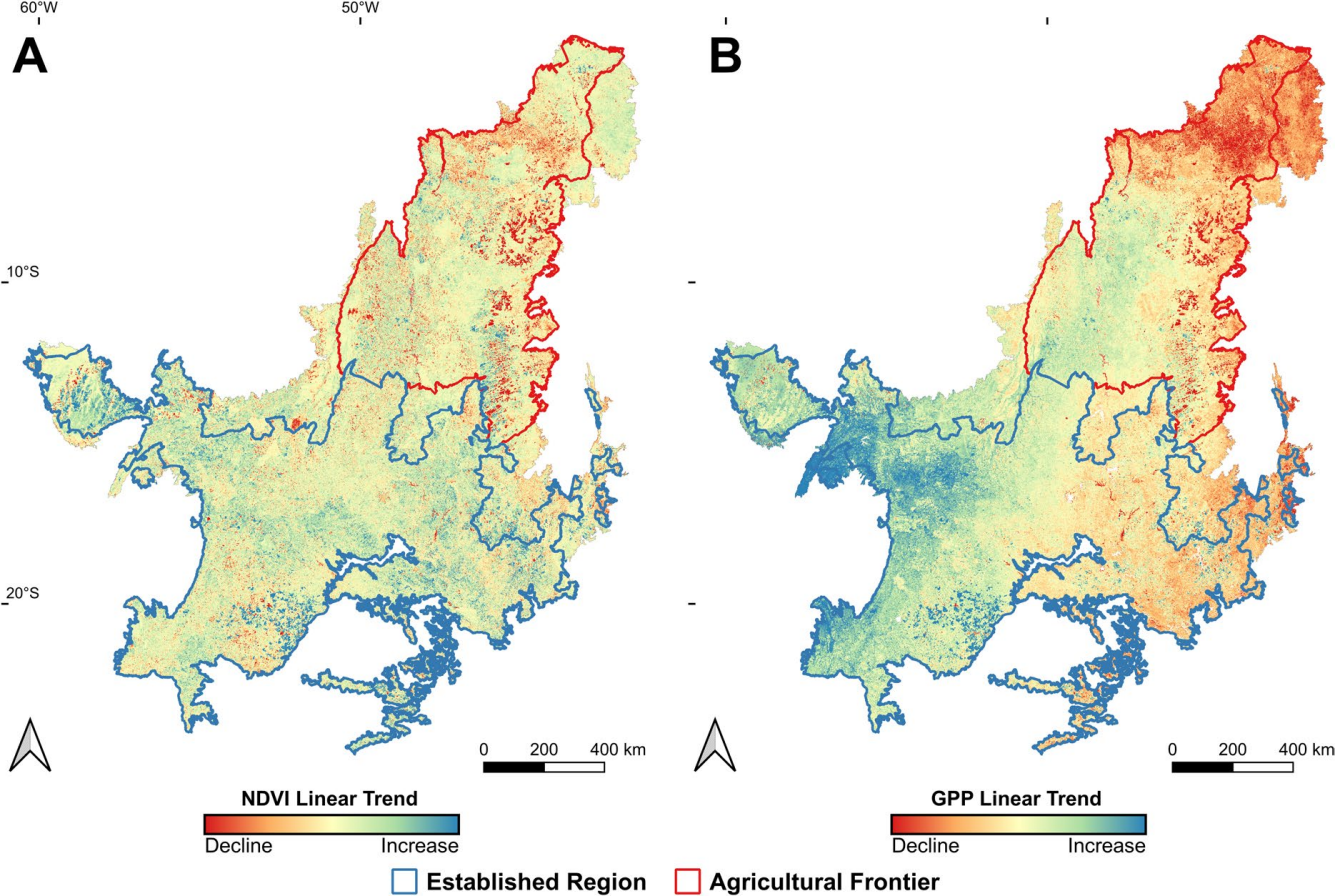
Long-term linear trends of MODIS **A** NDVI and **B** GPP across the established agricultural region (blue outline) and the emerging agricultural frontier (red outline) in the Brazilian Cerrado biome. The slope maps were derived from Trends.Earth analysis in the 2000–2022 period. Red colors indicate long-term declines in vegetation greenness and productivity, whereas blue colors indicate increasing trends. Yellow represents NDVI and GPP stability over time

Distinct long-term linear trends in MODIS NDVI (Fig. 4A) and MODIS GPP (Fig. 4B) were evident between the established agricultural region and the emerging frontier in the Cerrado biome. In the established region, areas of stable or increasing NDVI predominated, whereas the agricultural frontier exhibited a comparatively higher proportion of pixels with declining NDVI trends, as indicated by the red colors in Fig. 4A. Consistent with the NDVI patterns, the proportion of pixels exhibiting declining GPP trends was higher in the agricultural frontier, with large patches of reduced vegetation productivity concentrated in the northern part of MATOPIBA (red colors in Fig. 4B).

**Fig. 5:**
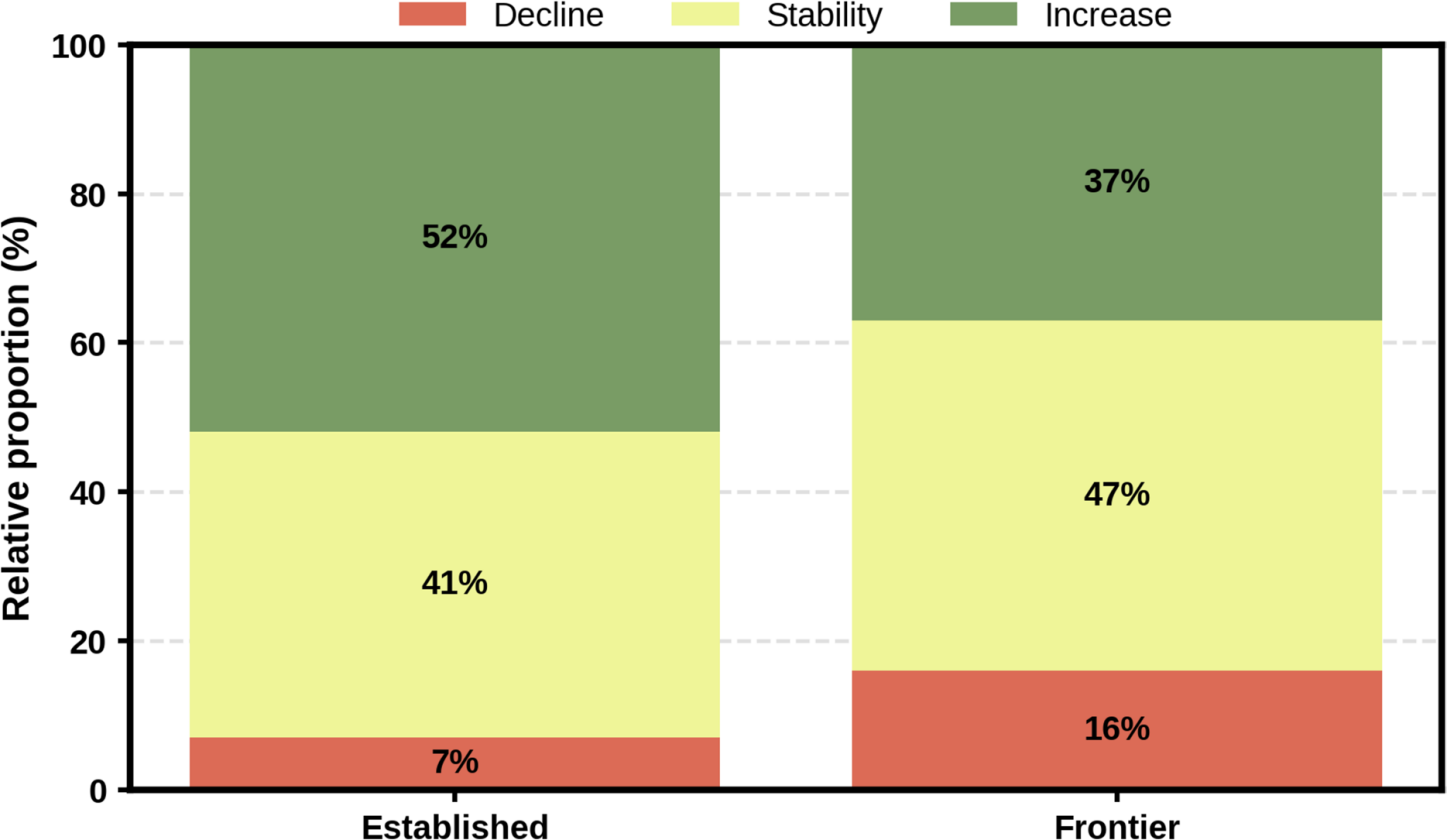
Relative proportions of pixels showing MODIS NDVI decline, stability, and increase in the established agricultural region and the agricultural frontier, during the 2000–2022 period of Trends.Earth analysis

The results shown in Fig. 4A revealed a remote-sensing paradox. The established agricultural region, where most native savanna clearing predated satellite observations and higher levels of land degradation would be expected, was the most stable. From Trends.Earth analysis, the established region (total area = 1,017,484 km^2^) exhibited the smallest proportion of pixels with declining NDVI (7%; 75,858 km^2^) and the highest proportion with increasing NDVI (52%; 528,542 km^2^) (Fig. 5). In contrast, the agricultural frontier (total area = 633,271 km^2^), where native savanna vegetation predominates and vegetation clearing largely coincided with the satellite observation period, showed a higher proportion of NDVI decline (16%; 103,188 km^2^) and a lower proportion of increase (37%; 232,459 km^2^) (Fig. 5). The proportion of pixels exhibiting NDVI stability ranged from 41% (413,084 km^2^) in the established region to 47% (297,624 km^2^) in the agricultural frontier.

**Fig. 6:**
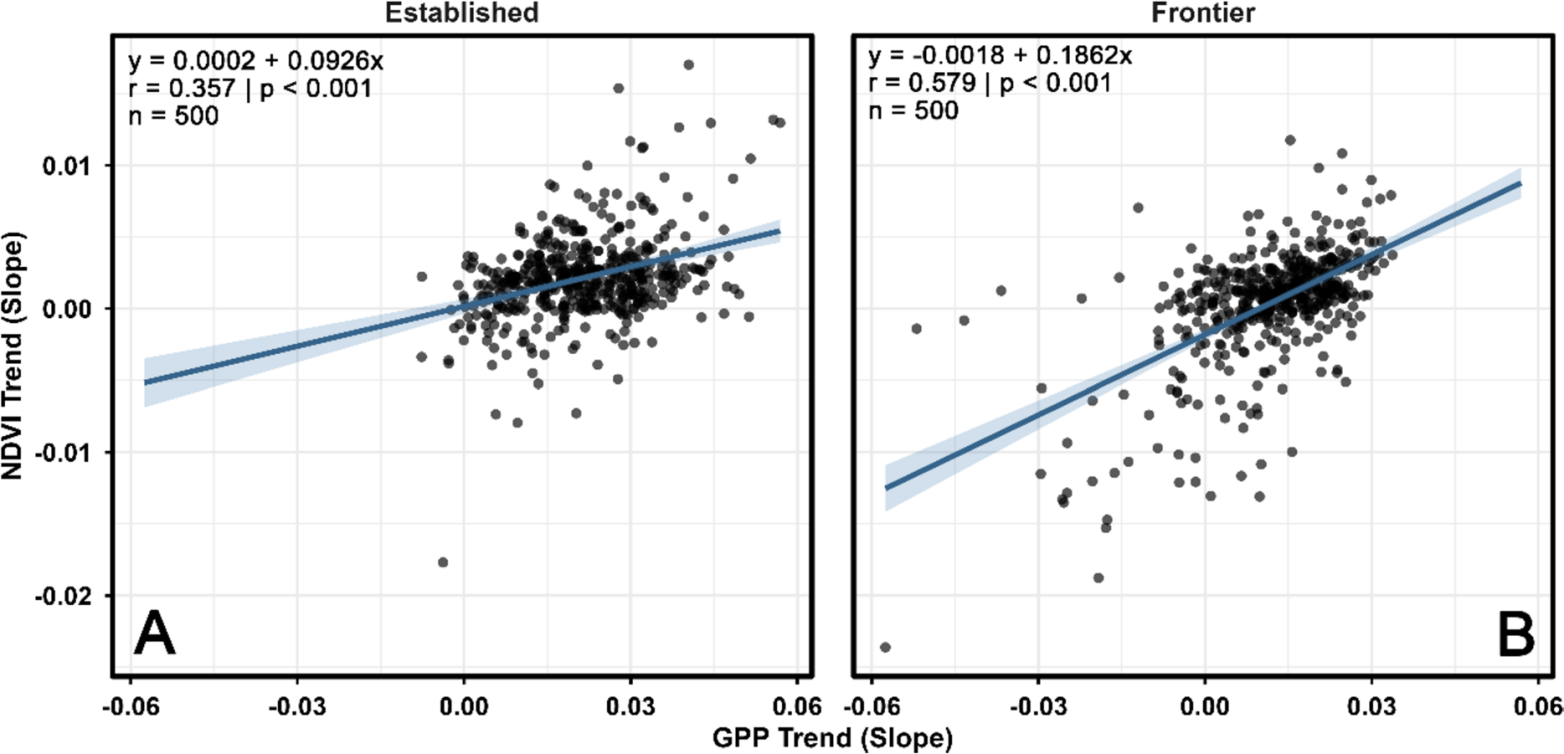
Relationships between linear trends of NDVI and GPP are shown for **A** the established region and **B** the agricultural frontier. Pearson’s correlation coefficients (*r*) are indicated at the 0.05 significance level, with confidence intervals shown around the fitted lines

Linear trends of MODIS NDVI and GPP showed positive associations (Fig. 6). Pearson’s correlation coefficients at the 0.05 significance level varied from +0.357 in the established region (Fig. 6A) to +0.579 in the agricultural frontier (Fig. 6B). Thus, although NDVI and GPP trends moved in the same direction in both regions, the relationship was weaker in the established region, explaining only a limited proportion of the variance in GPP trends. This weaker association suggests that, in consolidated agricultural landscapes, NDVI may not fully capture vegetation productivity dynamics, reflecting also region-specific land-use patterns. This result is consistent with the broader paradox observed in the established region, which is characterized by consolidated agricultural management and lower variability in NDVI and GPP trends (Fig. 6A). In contrast, the agricultural frontier showed greater dispersion in linear NDVI and GPP trends (Fig. 6B) and a stronger positive association, with relatively more declines, likely reflecting more recent land-cover transitions observed by the satellite and more pronounced changes in vegetation productivity.

### Drivers of MODIS NDVI trends by region

**Fig. 7:**
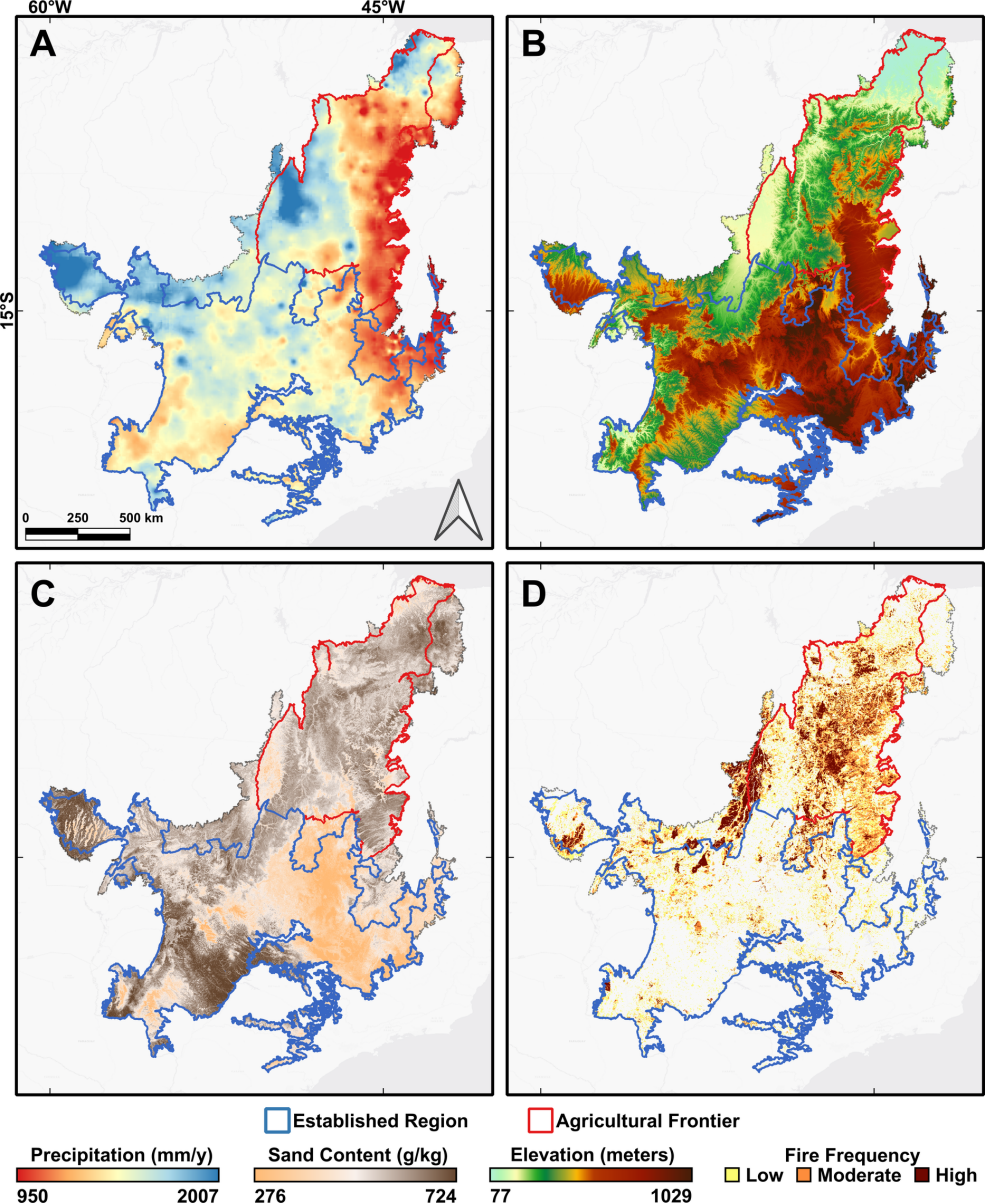
Differences between the established agricultural region (blue outline) and the agricultural frontier (red outline) in **A** mean annual precipitation, **B** elevation, **C** soil sand content, and **D** fire frequency. Results for **A** and **D** refer to the 2000–2022 period of analysis

The contrasting NDVI trajectories shown in Fig. 4 between the established agricultural region and the emerging frontier may be influenced by several factors listed in Table 1. For example, compared to the established region, the agricultural frontier experienced lower mean annual precipitation between 2000 and 2022, particularly in the eastern and southeastern portions near the semi-arid Caatinga biome (Fig. 7A). The frontier also has lower-elevation terrains, with some plateaus observed in the southeastern region (Fig. 7B). In addition, soil sand fractions are higher and more homogeneously distributed in the frontier than in the established region (Fig. 7C). The incidence of fire during the studied period is another notable difference, being comparatively higher in the frontier (Fig. 7D). All of these factors may contribute, to some extent, to the observed NDVI trajectories in each region, which requires statistical analysis for proper interpretation.

**Fig. 8:**
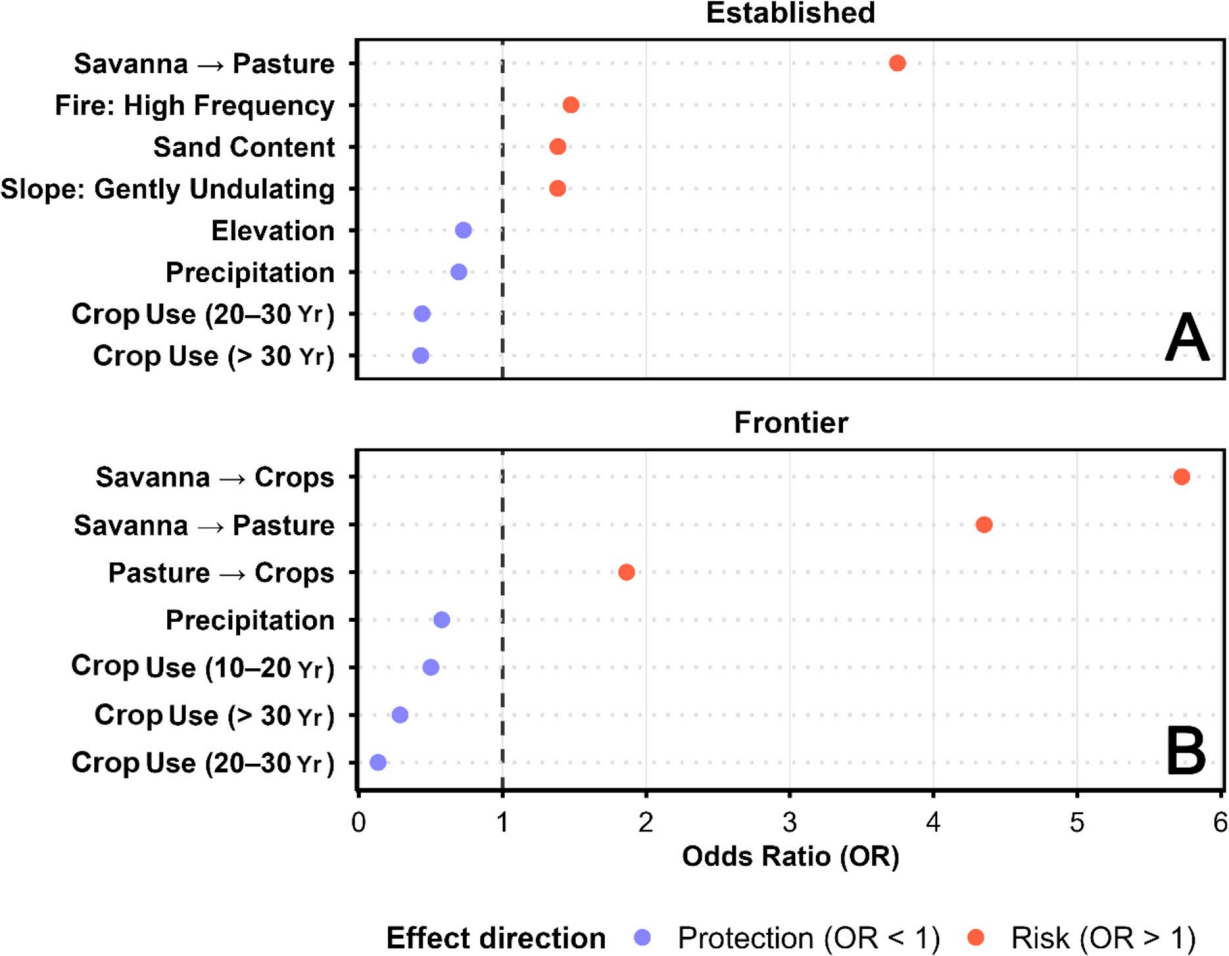
Main drivers of long-term MODIS NDVI decline in **A** the established agricultural region and **B** the agricultural frontier, based on odds ratios (OR) estimated from binary logistic regression. Red points indicate factors that increase the probability of NDVI decline (risk; OR > 1), while blue points indicate factors that reduce the probability (protection; OR < 1). The vertical dashed line at OR = 1 represents no effect

In this context, when applied separately to each region and to the variables listed in Table 1, binary logistic regression identified the most statistically significant drivers of long-term NDVI trends (Fig. 8). In both models, the response variable was the binarized Aggregated NDVI Trend Indicator, in which 1 represents long-term NDVI decline and 0 represents no decline. Based on odds ratios (OR) greater than 1, the key factors increasing the probability of NDVI decline in the established region were the conversion of native savanna vegetation to pastures, the fire frequency, the soil sand fraction, and the topographic slope, as indicated by the red symbols (risk in effect direction) in Fig. 8A. In the agricultural frontier, the primary drivers of NDVI decline were the conversion of native savanna into croplands and pastures, as well as the conversion of pastures into croplands (Fig. 8B). In both regions, higher precipitation and longer agricultural land use reduced the probability of NDVI decline, as indicated by the blue symbols in Fig. 8A and B. The duration of crop use likely reflects the implementation of soil and crop management practices that mitigate land degradation, thereby enhancing vegetation productivity and crop yields. Elevation was also an important driver in the frontier, likely reflecting the productive agricultural areas locally concentrated on southeastern plateaus.

The logistic regression models exhibited moderate discriminative performance. The agricultural frontier achieved slightly better results (accuracy = 73%, Kappa = 0.46, AUC = 0.796) than the established region (accuracy = 67%, Kappa = 0.34, AUC = 0.735), likely reflecting the inherent complexity of the Cerrado, the influence of omitted variables, and the data noise. The McNemar test (*p* > 0.70) did not indicate systematic classification bias, suggesting a balance between false positives and false negatives, although this does not imply high overall accuracy. Consequently, the reported ORs should be interpreted as indicators of probabilistic trends within a highly heterogeneous spatial context, rather than as deterministic predictions.

A detailed examination of the statistical results indicated that factors associated with an increased likelihood of NDVI decline exerted a weaker relative impact in the established region than in the agricultural frontier, as shown in the last columns of Tables 2 and 3, respectively. For example, the conversion of savannas to pastures resulted in a +275% increased risk of NDVI decline in the established region (Table 2), whereas the conversion of savannas to croplands produced a much stronger effect (+473%) in the agricultural frontier (Table 3). Among the factors reducing the probability of NDVI decline, the different classes of crop use duration showed reduced risks of −56% and −57% in the established region (Table 2), while in the agricultural frontier their impacts ranged from −50% to −87% (Table 3).

**Table 2:** Binary logistic regression results for the established agricultural region. The coefficient estimates (Estimate, in log-odds), standard error (Std. Error), z-statistic, significance (p), odds ratio (OR), and percentage impact are shown for the significant predictors with the strongest risk effects (OR > 1) and protective effects (OR < 1)

Variable	Estimate	Std. error	z	*p*	*OR*	% Impact
*Factors increasing likelihood of NDVI decline*						
Savanna conversion to pasture	1.32	0.62	2.13	0.033	3.75	+275
Fire frequency (High)	0.39	0.15	2.52	0.011	1.48	+48
Soil sand content	0.33	0.04	8.04	<0.001	1.39	+39
Slope (Gently undulating)	0.32	0.07	4.88	<0.001	1.38	+38
Fire frequency (Moderate)	0.29	0.08	3.61	<0.001	1.34	+33
*Factors reducing likelihood of NDVI decline*						
Crop use duration (>30 years)	−0.84	0.15	−5.81	<0.001	0.43	−57
Crop use duration (20–30 years)	−0.82	0.12	−6.81	<0.001	0.44	−56
Precipitation	−0.36	0.04	−9.81	<0.001	0.70	−30
Elevation	−0.32	0.04	−7.46	<0.001	0.73	−27
Crop use duration (10–20 years)	−0.31	0.10	−3.07	0.002	0.73	−27

**Table 3:** Binary logistic regression results for the agricultural frontier. The coefficient estimates (Estimate, in log-odds), standard error (Std. Error), z-statistic, significance (p), odds ratio (OR), and percentage impact are shown for the significant predictors with the strongest risk effects (OR > 1) and protective effects (OR < 1)

Variable	Estimate	Std. error	z	*p*	*OR*	% Impact
*Factors increasing likelihood of NDVI decline*						
Savanna conversion to crops	1.75	0.24	7.19	<0.001	5.73	+473
Savanna conversion to pastures	1.47	0.26	5.72	<0.001	4.35	+335
Pasture conversion to crops	0.62	0.25	2.46	0.014	1.86	+86
*Factors reducing likelihood of NDVI decline*						
Crop use duration (20–30 years)	−2.01	0.13	−15.90	<0.001	0.13	−87
Crop use duration (>30 years)	−1.25	0.25	−5.01	<0.001	0.29	−71
Crop use duration (10–20 years)	−0.69	0.08	−8.86	<0.001	0.50	−50
Precipitation	−0.55	0.05	−11.69	<0.001	0.58	−42
Fire frequency (High)	−0.44	0.11	−3.91	<0.001	0.64	−36
Pasture use duration (20–30 years)	−0.44	0.13	−3.52	<0.001	0.64	−36
Elevation	−0.27	0.04	−5.94	<0.001	0.77	−23
Pasture use duration (10–20 years)	−0.23	0.10	−2.25	0.024	0.80	−20

The results presented in Fig. 8 and Tables 2 and 3 indicate that the main driver of NDVI decline in each region is the conversion of native savanna vegetation to other land covers. The dynamics of this conversion were previously illustrated in Fig. 3. On the other hand, the duration of agricultural land use, likely reflecting the effects of soil, pasture, and crop management practices in increasing vegetation productivity, emerged as shared primary drivers of NDVI increase. Secondary drivers of NDVI decline included fire frequency, soil sand fractions, and local topographic conditions. In contrast, secondary drivers of NDVI increase were mostly related to precipitation and topographic elevation favoring agricultural activity.

### Common and region-specific drivers of MODIS NDVI trends

To identify common and region-specific drivers of long-term MODIS NDVI trends, results of the Wald-type Z test are summarized in Table 4. They are used to assess the statistical equality of the estimate coefficients for the logistic regression models obtained from the established region and the agricultural frontier. Based on this criterion, variables were categorized as common factors (statistically equivalent effects between regions) and non-common factors (effects with statistically significant differences). Only variables that were statistically significant in both models are presented in Table 4.

**Table 4:** Common and region-specific significant drivers of NDVI trends for variables that were statistically significant in both models, together with their corresponding odds ratios (OR). Variables are grouped into common factors (no significant difference between regions) and non-common factors (significant differences), based on a Wald-type Z test for coefficient comparison

Variable	OR (established region)	OR (Frontier)	Significant difference
*Common factors between regions*			
Savanna conversion to pasture	3.75	4.35	NO (*p* = 0.82)
Savanna conversion to crops	3.02	5.73	NO (*p* = 0.31)
Elevation	0.73	0.77	NO (*p* = 0.39)
Crop use duration (>30 years)	0.43	0.29	NO (*p* = 0.15)
*Non-common factors between regions*			
Precipitation	0.70	0.58	YES (*p* < 0.001)
Fire frequency (High)	1.48	0.64	YES (*p* < 0.001)
Crop use duration (10–20 years)	0.73	0.50	YES (*p* = 0.003)
Crop use duration (20–30 years)	0.44	0.13	YES (*p* < 0.001)

Four common drivers of NDVI trends across regions were identified from Wald-type Z tests, indicating similar dynamics in affecting the probability of NDVI decline (savanna conversion to croplands and to pastures) or NDVI increase (elevation and the duration of crop use superior to 30 years). As shown in the last column of Table 4, no statistically significant differences were recorded for them during the comparison of the regression estimate coefficients generated for each region. While the removal of native vegetation in favor of croplands and pastures represents a universal driver of long-term NDVI decline, the presence of productive croplands on plateaus and the effects of agricultural management associated with longer land use tend to promote NDVI trend increase.

Non-common drivers of NDVI trends across regions included precipitation, fire frequency, and two classes of crop use duration (Table 4). These region-specific drivers of NDVI trajectory differed in both the intensity and, in some cases, the direction of their effects. For example, mean annual precipitation reduced the probability of NDVI decline in both regions, but with a significantly stronger effect in the agricultural frontier, as indicated by the lower odds ratio in Table 4. This finding is consistent with Fig. 7A, which shows that the agricultural frontier encompasses the driest areas of the Cerrado biome, adjacent to the semi-arid Caatinga biome in the eastern region, where water limitation is more pronounced. Another example is high fire frequency, which increased the probability of NDVI decline in the established region while reducing the likelihood of decline in the agricultural frontier (Table 4). The possible reasons for this contrasting pattern are discussed in the next section.

Overall, relative to the established region, the agricultural frontier exhibited NDVI decline more strongly linked to LULC changes, reflecting an active phase of agricultural expansion and landscape reorganization.

## Discussion

This investigation constitutes the first large-scale study of vegetation index trends in the Brazilian savanna environment, comparing two contrasting regions of the Cerrado with distinct land-use history and climatic/environmental conditions. The results revealed a remote-sensing paradox in long-term MODIS NDVI trends after comparison of the established agricultural region with the newest agricultural frontier (MATOPIBA). In both regions, NDVI declines were primarily driven by the conversion of savanna vegetation to croplands and pastures, with stronger impacts observed in the frontier, as indicated by the statistical analysis. Paradoxically, the established region, where most clearing occurred before satellite observations, was more stable, exhibiting smaller pixel proportions of NDVI declines (7%) and larger pixel proportions of NDVI increase (52%) than the agricultural frontier (16% and 37%, respectively). These patterns indicate that the interpretation of declines and increases in the NDVI trends detected by orbital sensors critically depends on the timing of land-use occupation relative to the satellite observation period, or the definition of the baseline of landscape change. Tracking land-use history is critical for defining this baseline and for accurately interpreting NDVI trends as ecological indicators of land degradation at the biome scale.

In the established region, earlier vegetation conversion and subsequent soil, pasture, and crop management practices likely masked previous land degradation processes from spectral detection. As reported in the literature, these practices, including fertilization, soil acidity correction, crop rotation, regenerative agriculture, improved crop varieties, and crop–livestock–forest integration, enhance soil and crop conditions and increase crop yield (Muhie, 2022; Souza et al., 2024). They increase vegetation productivity and NDVI trends. Consistently, the aggregated effect of these practices was indirectly captured in our models by the duration of agricultural use, which emerged as one of the main drivers associated with a reduced likelihood of NDVI decline (i.e., greater NDVI stability or a tendency of increase). This interpretation aligns with Trabaquini et al. (2015), who have indicated the absence of direct relationship between duration of agricultural land use and soybean yield in the Cerrado. They suggested that agricultural management, not time itself, is the underlying mechanism sustaining crop yield. Consistent with our findings, Shrestha et al. (2024) highlighted the significant role of anthropogenic activities in explaining MODIS NDVI variability from 2003 to 2022 in Nepal when studying other vegetation types.

Additionally, we observed that NDVI trends were positively correlated with MODIS GPP in both regions, supporting the use of NDVI as a proxy for vegetation productivity (Wang et al., 2004). However, the magnitude of the relationships differed across regions. Consistent with the broader paradox argument, correlations between NDVI and GPP trends were weaker in the established region than in the agricultural frontier, likely reflecting the lower variability in trend magnitude associated with more consolidated agricultural activities. In this context, our results underscore the need for caution when interpreting NDVI trends as a proxy for land degradation in the Cerrado. Although the conversion of native savanna vegetation to agriculture constituted a common driver of NDVI decline across regions, the satellite-observed signal may predominantly reflect changes in vegetation derived from agricultural management practices adopted to increase vegetation productivity. This is especially true in the established agricultural region where the critical vegetation conversion phase occurred prior to the analyzed satellite period.

A previous study in MATOPIBA highlighted the need for further investigation into the links among vegetation productivity, land degradation, precipitation, sandy soils, and fire frequency (Souza et al., 2020). In this sense, our experimental design contributes to improved understanding of the drivers controlling the NDVI trajectories by modelling several potential factors such as LULC changes, duration of pasture and cropland use, fire frequency, precipitation, soil sand fraction, and topography. The logistic regression and Wald-type tests identified the conversion of native Cerrado vegetation into croplands and pastures as common primary drivers of NDVI decline across regions. Elevation and agricultural land use exceeding 30 years contributed to the NDVI increase. Region-specific drivers of NDVI variability, such as soil sand fraction, precipitation, and fire frequency, accounted for the additional regional variability. Thus, we confirmed our hypothesis that NDVI trajectories in the Cerrado exhibit distinct regional patterns, driven in part by a combination of shared and region-specific factors shaping NDVI trends across areas with contrasting land-use histories.

Given the climatic and environmental differences between the established agricultural region and the expanding frontier, the capture of other region-specific drivers in the statistical modelling was expected. For instance, the agricultural frontier exhibits higher and more homogeneously distributed soil sand fractions than the established region. Eastern portions of the frontier, particularly those near the semi-arid Caatinga biome in northeastern Brazil, are drier and dominated by sandy soils (Araújo et al., 2019). Consistently, soil texture and composition have been identified as key local drivers of pasture degradation in a microregion of the Brazilian state of Goiás (Silva et al., 2025), with other studies similarly linking degradation to weakly structured sandy soils (Vieira et al., 2021). The agricultural frontier also exhibits substantially higher fire frequency than the established region. Our model indicates that high fire frequency increases the probability of NDVI decline in the established region but is associated with lower odds of decline in the agricultural frontier. This reversal trend should be interpreted with caution, as it does not necessarily indicate a direct protective effect of fire. Instead, it likely reflects the complex interplay among fire occurrence, land-cover composition, and post-disturbance vegetation dynamics. In MATOPIBA, fire is mainly associated with vegetation clearing and land-management practices related to the expansion of soybean cultivation and pasture, which may contribute to NDVI decline. However, areas with high fire frequency in the agricultural frontier can also include native savanna formations, where fire is part of the ecological dynamics of the ecosystem. In these areas, fire can alter savanna structure and composition, with effects that vary among physiognomies and depend on fire frequency and severity (Miranda et al., 2009). For instance, savanna grasslands may recover rapidly after burning and sometimes exhibit higher NDVI values than unburned areas during the seasonal cycle following disturbance (Santos et al., 2025). Therefore, the lower odds of NDVI decline associated with high fire frequency in the agricultural frontier should be interpreted as a signal likely shaped by several factors such as post-fire vegetation recovery, savanna physiognomies, and the greater extent of preserved native vegetation compared to the established region.

The current LULC analyses provided additional support for interpreting the regional asymmetry of NDVI trajectories. In the established agricultural region, land occupation from 1985 to 2022 occurred in two distinct phases, as indicated by our results. The first (1985–2005) was driven by the soybean boom in the savanna, initiated in the 1970 s through policies promoting land-use intensification and soybean cultivation, mainly from south to north across the region (Lima et al., 2019; Santos, 2019). From our findings, this phase led to the clearance of approximately 65% of the original savanna vegetation of the established region, with clearing rates stabilizing after 2005. From our results, as savanna conversion declined after 2005 in the established region, crop expansion increasingly occurred over existing pastures, consistent with observations from another study in southwest Goiás (Silva et al., 2025). In contrast, the agricultural frontier had cleared about 30% of its original vegetation cover by 2022, reflecting ongoing expansion of crops and pastures at high rates of savanna clearing. This expansion has been spatially heterogeneous across MATOPIBA (Souza et al., 2020). The agricultural expansion has been attributed to factors such as the spillover effects of the 2006 Amazon Soy Moratorium over the savanna clearing, land and commodity prices, agricultural credit policies, infrastructure development, and advances in soil and crop management practices (Araújo et al., 2019; Bispo et al., 2023; Bolfe et al., 2016; Mesquita, 2011; Spagnolo, 2011). Compared to the Amazonian tropical forests, the savannas from the Cerrado biome are legally more vulnerable to vegetation clearing (Lima et al., 2019). These contrasting regional trajectories help explain the stronger NDVI decline observed in the frontier. In MATOPIBA, most LULC changes occurred within the MODIS observation period, whereas in the established region the satellite data largely reflects later phases of land occupation dominated by agricultural intensification.

Thus, our findings indicate that long-term NDVI trajectories in the Cerrado should not be interpreted solely as direct indicators of land degradation. NDVI trajectories of decline or increase are shaped by a combination of factors, including LULC changes, land-use history, and agricultural management within the satellite observation period, as well as local environmental and climatic conditions. Consequently, interpreting NDVI trends as proxies for land degradation requires a contextualized approach that explicitly accounts for these interacting factors and the temporal window of the satellite time series.

Finally, it is important to acknowledge some limitations inherent to this study. Each remote sensing product presents its own uncertainties, which are propagated throughout the data analyses. Part of the explanatory variables were derived from datasets with differing spatial resolutions, which may introduce uncertainties associated with resampling processes and the spatial representation of the analyzed phenomena. The resulting scale mismatch is acknowledged as an inherent limitation with three main implications: (i) LULC transitions detected at 30 m may not be immediately reflected in the integrated spectral response of the 250 m pixel, artificially inflating the model’s statistical accuracy; (ii) the resolution of CHIRPS introduces a generalization bias by assuming uniform water availability at 5-km pixel size; and (iii) resampling procedures may generate artificial gradients through interpolation or omit relevant information through aggregation.

There are also limitations associated with the method used to correct for climatic variability. For example, RUE assumes a positive relationship between vegetation productivity and rainfall, which may not always hold in areas bordering the semi-arid Caatinga biome due to the spatial scale mismatch between CHIRPS data (~5 km resolution) and NDVI pixels (250 m). In addition, the influence of agricultural management on NDVI trajectories was not directly quantified, and the effects of vegetation productivity were not disentangled from land degradation, highlighting the need for future investigations based on independent biophysical and edaphic indicators, ideally combined with field measurements. Other land covers also strongly influenced NDVI trends but were excluded from the analysis due to their limited geographic occurrence. For example, pine plantations in the established region that grow over several years during the satellite observation period will exhibit increasing NDVI trends. If harvesting occurs during this period, NDVI declines will be observed. Therefore, long-term NDVI trends in planted forests depend on the timing of plantation and harvest relative to the satellite record.

Despite these constraints, the delineation of regions based on objective criteria, namely the duration of agricultural use and the remaining savanna cover, strengthened the consistency of biome-scale comparisons. The 20-year threshold was adopted to ensure that vegetation suppression predominantly occurred prior to the MODIS record (2000–2022), while acknowledging that alternative thresholds could lead to slight variations in the proportions of classified pixels, without, however, compromising the contrast between regions or the identified paradox. Although this approach entails additional methodological challenges, particularly regarding sensor harmonization, it represents a promising pathway to deepen understanding of land-use legacies on long-term vegetation trajectories in the Cerrado.

Future studies could explore the use of Landsat time series to fix a given region in the Cerrado while varying satellite observation periods across temporal windows preceding MODIS observations. This approach would be a variant of our experimental design and would benefit from Landsat’s higher spatial resolution. However, this strategy requires harmonizing time series across Landsat sensors with different technical specifications, which may be challenging for data acquisition preceding MODIS observations. An example of this strategy is the Harmonized Landsat and Sentinel-2 (HLS) product, unfortunately available for data since 2013. This product compensates for differences in factors such as bidirectional effects, bandwidth, and spectral band positioning (Ju et al., 2025).

## Conclusions

We identified a remote-sensing paradox between the established agricultural region and the agricultural frontier in the Brazilian Cerrado. The agricultural frontier exhibited higher fire frequency and greater soil sand content than the established region, along with lower precipitation in eastern MATOPIBA near the semi-arid Caatinga biome of northeastern Brazil. Although region-specific drivers influenced vegetation index trajectories, the dominant primary driver of long-term MODIS NDVI decline in both regions was the conversion of native savanna vegetation to croplands and pastures. Paradoxically, the established agricultural region, where most savanna clearing occurred prior to satellite observations, was the most stable, showing the smallest pixel proportion of NDVI decrease (7%; 75,858 km^2^) and the largest increase (52%; 528,542 km^2^). In contrast, the agricultural frontier, where native vegetation predominates and clearing largely coincided with the satellite record, exhibited a larger pixel proportion of NDVI decline (16%; 103,188 km^2^) and smaller proportion of increase (37%; 232,459 km^2^).

Binary logistic regression and Wald-type Z tests showed that key mechanisms of NDVI decline are shared across regions. Conversion of native savanna to pasture and croplands consistently increased the odds of NDVI decline, whereas elevation and long-established croplands (>30 years) promoted NDVI increases in both regions. Other secondary predictors acted as region-specific drivers of NDVI trajectories. For instance, precipitation reduced the probability of NDVI decline in the frontier, while soil sand content and high fire frequency had opposite effects between regions.

The duration of agricultural use contributed to NDVI increases, suggesting that soil and crop management practices mask underlying land degradation. Increasing NDVI trends were positively correlated with MODIS GPP, confirming NDVI as a proxy for vegetation productivity. However, contributing to the observed paradox, these relationships were weaker in the established region than in the emerging frontier. Thus, interpretation of NDVI trajectories as indicators of land degradation in the Cerrado requires explicit consideration of land-use history relative to the satellite observation period and the influence of agricultural management practices on long-term NDVI trends.

## Data Availability

Data will be made available on request.
